# Cross sectional study of prevalence, genetic diversity and zoonotic potential of *Cryptosporidium parvum* cycling in New Zealand dairy farms

**DOI:** 10.1186/s13071-015-0855-9

**Published:** 2015-04-22

**Authors:** Julanda Al Mawly, Alex Grinberg, Niluka Velathanthiri, Nigel French

**Affiliations:** mEpiLab, Hopkirk Research Institute, Massey University, Palmerston North, New Zealand; Infectious Diseases Group, Institute of Veterinary, Animal and Biomedical Sciences, Massey University, Private Bag 11-222, Palmerston North, New Zealand

**Keywords:** *Cryptosporidium*, Calves, Zoonosis, Diarrhea, Prevalence

## Abstract

**Background:**

The estimation of the prevalence and zoonotic potential of *Cryptosporidium parvum* cycling in bovine populations requires the use of genotyping, as several morphologically similar non-parvum genetic variants of unproven clinical and public health impact are found in cattle. However, robust *C. parvum* prevalence estimates in cattle are lacking and comparative data of bovine and human isolates collected from the same regions are scarce. Thus, the relative contribution of the *C. parvum* oocysts released by farmed animals to animal and human cryptosporidiosis burden is, in general, poorly understood.

**Methods:**

The New Zealand farm-level *C. parvum* prevalence was estimated using a cross-sectional sample of 1283 faecal specimens collected from newborn calves on 97 dairy farms. Faeces were analysed by immunofluorescence and the *Cryptosporidium* parasites were genetically identified. Finally, bovine *C. parvum* were genetically compared with historical human clinical isolates using a bilocus subtyping scheme.

**Results:**

Immunofluoresence-positive faeces were found in 63/97 (65%) farms. *C. parvum* was identified in 49 (50.5%) farms, *C. bovis* in 6 (6.1%) farms, and on 8 (8.2%) farms the species could not be identified. The dominant *C. parvum* genetic variants were geographically widespread and found in both host populations, but several variants were found in humans only.

**Conclusions:**

Phenotypic tests offered by New Zealand veterinary diagnostic laboratories for the diagnosis of *C. parvum* may have moderate to high positive predictive values for this species. The genetic similarities observed between the human and bovine parasites support a model considering calves as significant amplifiers of zoonotic *C. parvum* in New Zealand. However, data suggest that transmission routes not associated with dairy cattle should also be taken into account in future source-attribution studies of human cryptosporidiosis.

## Background

Protozoa belonging to genus *Cryptosporidium*, in particular the intestinal species *C. parvum* and *C. hominis,* are major causes of human diarrhoea worldwide [1]. Whereas *C. hominis* is predominantly found in humans, *C. parvum* cycles extensively also in young calves and is one of the most important agents of neonatal calf diarrhoea [2,3]. Furthermore, farmed cattle, especially newborn calves, might be a source of zoonotic *C. parvum* infections via direct transmission or indirectly, through the deposition of faecal material into water sources or agricultural land [4].

Until the early 2000s, when round *Cryptosporidium* oocysts were identified in calves’ faeces, they were collectively classified as *C. parvum* and considered pathogenic and potentially zoonotic. Furthermore, the mere presence of *C. parvum* infections in humans was generally viewed as an indication of the occurrence of zoonotic cryptosporidiosis in the region [4-6]. This situation started to change with the use of genotyping in epidemiological research, which led to the discovery of new *Cryptosporidium* taxa in cattle such as *C. bovis,* and of ‘anthroponotic’ *C. parvum* lineages that are not found in cattle [7-9]. In addition to *C. parvum*, the following taxa producing round, indistinguishable oocysts have so far been identified in calves: *C. bovis* [7], *C. ryanae* (formerly the ‘deer-like’ genotype) [10], *C. ubiquitum* (formerly the ‘cervine genotype’) [11], *C. suis, C. scrofarum* [12,13], and *C. hominis* [14]. Whereas *C. parvum* is a frank pathogen of calves, the clinical significance and zoonotic impact of the other taxa is not established. Thus, assessing the prevalence and genetic diversity of *C. parvum* cycling in cattle populations is important from both animal and public health perspectives.

Epidemiological studies of *Cryptosporidium* in cattle applying genotyping have been performed in different regions using non-random, mostly opportunistic samples, or faecal specimens submitted to diagnostic laboratories [5,8,15,16]. Surprisingly, however, epidemiologically robust estimates of *C. parvum* prevalence in cattle populations extrapolated using representative random samples are lacking, and genetic comparisons of bovine and human parasites isolated in the same region are very scarce [8]. Therefore, the impact of *C. parvum* oocysts originating from cattle farms on human cryptosporidiosis burden is, in general, poorly understood.

New Zealand is a major dairy producer^a^. Here, human cryptosporidiosis is a notifiable condition, and a notification system underpinned by genotyping efforts is in place. Most New Zealand dairy farms manage short calving seasons between July and October, and human infections follow a regular pattern, with *C. parvum* notifications peaking every year during, or soon after the calving season^b^. Yet, the relative contribution of the oocysts originating from dairy farms on human cryptosporidiosis burden is not known as the genetic diversity of *C. parvum* cycling in cattle has not been studied in detail and comparative molecular data on human and bovine isolates are scarce.

New Zealand operates a strict biosecurity system, with limited importation of livestock or goods posing a risk for the national herd. This close ecosystem presented an opportunity to assess the genetic diversity of the endemic *C. parvum* of dairy cattle. In this paper, we report the results of a study aimed at estimating the New Zealand dairy farm-level prevalence and the genetic diversity of bovine *C. parvum*. The availability of the sequences of a large number of historical human clinical isolates allowed a genetic comparison of bovine and human C*. parvum* at national and regional levels, enhancing our understanding of the epidemiology of zoonotic cryptosporidiosis. The results indicate a *C. parvum* farm-level prevalence of 50.5% and a high degree of genetic similarity between human and bovine isolates*,* supporting a model that considers newborn calves as significant amplifiers of potentially zoonotic parasites*.* Nonetheless, evidence for the occurrence of human infections unrelated to the dairy cattle reservoir is also provided.

## Methods

### Study design and sampling

This study used faecal specimens collected from calves on 97 New Zealand dairy farms, as part of a project assessing the national prevalence of enteropathogens and risk factors for neonatal calf diarrhoea. The sampling frame consisted in a national register of dairy farms [17], and the ethical approval and random sampling of farms has been previously described [18]. Briefly, the sampling was performed during the second half of the winter calving season, between July and October 2011. The target population was that of newborn calves present on New Zealand farms milking more than 150 cows. This minimum herd-size was targeted in order to sample multiple calves at the peak of shedding on each farm, enhancing farm-level testing regime sensitivity. There were approximately 10,600 eligible farms in the register, corresponding to 88% of the total number of farms in the country^a^. Five North Island (Waikato, Wellington, Northland, Taranaki and Manawatu-Wanganui) and two South Island regions (Canterbury and Southland) were selected based on their high density of dairy cattle. Collectively, these regions included 75% of the eligible farms in the database. A tentative target sample size of 120 farms was determined based on the maximum number of farms that could be reached during the second half of the calving season. Each recruited farm was visited once, and in order to cover the required spectrum of enteropathogens, farmers were asked to facilitate the sampling of 1 to 5-day-old and 9 to 21-day-old calves present on the farm (the second age group was predicted to be at the peak of *C. parvum* shedding; [18]). After accounting for mortality and culled calves, in a hypothetical 60-day calving season a farm milking 150 cows would have provided ~15 calves for sampling. Assuming a calf-level *Cryptosporidium* test sensitivity of 0.7 and an infection rate on the day of sampling as low as 20%, a sample of 15 calves provided a farm-level testing sensitivity^c^ of 0.973 (specificity confirmed by genotyping, thus assumed to be 100%).

Samplers collected ~10 g of faeces from the rectums of the calves presented for sampling, changing disposable gloves between animals. Faecal samples were delivered to Massey University (MU) overnight and stored at 4 ± 2°C until analysed.

### Analysis for *Cryptosporidium* oocysts and genotyping of parasites

Analysis for *Cryptosporidium* oocysts in faces was performed by means of direct immunofluorescence staining (IFA) using a commercial kit (Aqua-Glo G/C, Waterborne Inc., New Orleans, USA). For species identification, 100 IFA-positive specimens selected in a blind fashion were genotyped by PCR-sequencing of the *Cryptosporidium* 18S ribosomal RNA gene (18S rDNA). At least one specimen per IFA-positive farm was genotype. Primers were 5-GTTAAACTGCGAATGGCTCA-3 (forward) and 5-CCATTTCCTTCGAAACAGGA-3 (reverse). The forward primer’s 5-end started at position 80 of the *C. parvum* 18S rDNA sequence [GenBank: AF108865.1] and annealed to *C. parvum*, *C. bovis* and *C. hominis*. The complement of the reverse primer’s 3-end started at position 1471 of the positive strand of the same sequence and annealed to *C. parvum*, *C. bovis*, *C. hominis* and *C. ryanane* (at the time of this analysis annealing to other *Cryptosporidium* found in calves was not known). Amplification was performed in a volume of 20 μl containing 2 μl 10 × PCR buffer, 1 μl dNTP (2 mM), 1 μl MgCl_2_ (50 mM), 2 μl non-acetylated bovine serum albumin (2 mg/ml) (New England Biolabs, USA), 4 pmoles of each primer and 0.5 μl (2.5 units) of *Taq* polymerase (Platinum® *Taq* DNA Polymerase, Invitrogen Corporation, Carlsbad CA, USA). The reaction was performed with initial denaturation at 96°C for 2 min, followed by 40 cycles at 94°C for 30 sec, 55°C for 30 sec and 72°C for 30 sec. Products were purified using an in-house ethanol purification protocol and the expected amplicon verified by 1% agarose gel electrophoresis. Bidirectional sequencing of an internal segment was performed using primers 5- CTCGACTTTATGGAAGGGTTG-3 (forward) and 5-CCTCCAATCTCTAGTTGGCATA-3 (reverse). The forward sequence annealed to *C. hominis* and *C. parvum* and differed by three nucleotides from *C. bovis*. The reverse sequence aligned to *C. parvum*, *C. bovis*, *C. hominis C. ryanae*. Forward and reverse sequences were aligned and edited manually using software (Geneious version 5.6.5; Biomatters Ltd., Auckland, New Zealand). Sequence ends that could not be edited were trimmed and the final sequences searched in the GenBank using Blast^d^.

Identified *Cryptosporidium* were subtyped using a bilocus genotyping scheme based on PCR-sequencing of the polymorphic regions of the single copy 60 kDa zoite glycoprotein (gp60) and the 70 kDa heat shock protein (Hsp70) genes [19,20]. These have been found to be among the most polymorphic loci in a previous large scale multilocus genotyping study and used for *C. parvum* subtyping in New Zealand [8,21]. The gp60 was also advantageous as this locus is widely used internationally for *C. parvum* subtyping, underpinning comparisons between studies.

The gp60 locus spanned an imperfect TCA/TCG repeat encoding a homoserine displaying length and repeat motif polymorphism. Amplification was performed using a nested PCR. The 20 μl outer reaction contained 2 μl 10 × PCR buffer, 1 μl dNTP (2 mM), 1 μl MgCl_2_ (50 mM), 2 μl non-acetylated bovine serum albumin (2 mg/ml) (New England Biolabs, USA), 4 pmoles each of primers 5-ATAGTCTCCGCTATATTC-3 (forward) and 5-GGAAGGAACGATGTATCT-3 (reverse), and 0.5 μl *Taq* polymerase (Platinum® Taq DNA Polymerase; Invitrogen Corporation, Carlsbad CA). Amplification was carried out with initial denaturation at 96°C for 2 min, followed by 40 cycles at 94°C for 20 sec, 57°C for 20 sec, and 72°C for 30 sec., with a final extension at 72°C for 10 sec. Two μl of the outer products were used as template for the inner PCR, which was run using the same reagents except for 0.6 μl MgCl_2_ (50 mM). The primers used in the inner PCR were 5-TCCGCTGTATTCTCAGCC-3 (forward) and 5-GCAGAGGAACCAGCATC-3 (reverse). Products were purified and sent for bidirectional Sanger-sequencing using the inner primers and raw sequences were edited as above. Multiple alignments were performed using edited sequences and the gp60 alleles were designated according to the nomenclature proposed by Sulaiman *et al*. [22]. For instance, the IIaA18G3R1 allele belongs to the *C. parvum* subtype family ‘IIa’ contains 18 TCA, three TCG repeats and one ACATCA terminal sequence.

The amplified Hsp70 locus spanned an imperfect repeat composed of a variable number of synonymous 12-base-long units coding a GGMP amino-acid sequence [20]. A search in GenBank identified *C. parvum* Hsp70 sequences coding 10, 11 and 12 GGMP units, worldwide (eg. GenBank: EU141724; KC823128.1; KC823127). Sequencing of the Hsp70 locus enhanced discriminatory power compared to fragment analysis due to the presence of synonymous repeat variants of the same length. Primers were 5-CACCATCCAAGAACCAAAGG-3 (forward) and 5-GCCTAAAGGTAGAGTGTGCTTTTC-3 (reverse) (a search in GenBank returned the forward primer’s sequence in isolates reported as *C. parvum*, *C. hominis*, *C. erinacei, C. viatorum*, *C. cuniculum* and *C. wrairi,* and the reverse primer in *C. parvum* only). Amplification was carried out in 20 μl containing 2 μl 10x PCR buffer, 1 μl dNTP (2 mM), 0.6 μl MgCl2 (50 mM), 2 μl non-acetylated bovine serum albumin (2 mg/mL, 200nM of each primer and 0.5 μl of *Taq* polymerase. Thermocycling included initial denaturation at 94°C for 2 min followed by 40 cycles at 94°C for 20 sec, 57°C for 20 sec and 72°C for 20 sec. Amplicons were verified by agarose electrophoresis, purified using an in-house ethanol purification method and sent for sequencing with the same primers. Raw sequences were edited as above.

Sequencing was performed by the Massey University Genomic Service (Palmerston North, New Zealand).

### Analysis of data

#### Calculation of prevalence and assessment of *C. parvum* genetic diversity

The crude *C. parvum* farm-level prevalence was computed as the number of farms providing at least one *C. parvum-*positive specimen confirmed by 18S rDNA sequencing, divided by the number of sampled farms.

The bilocus genotype (BLG) of each bovine isolate was designated using the combination of gp60 and Hsp70 alleles identified by sequencing. For instance, the IIaA18G3R3/11 includes the IIaA18G3R3 gp60 allelle and an Hsp70 sequence containing 11 GGMP-coding units, and the IIaA18G3R3/11_v1_ includes the IIaA18G3R3 and a synonymous Hsp70 variant composed of 11 GGMP-coding units.

The total number of gp60 allele types occurring in bovine *C. parvum* in New Zealand was estimated using the Chao1 taxonomic richness estimator and its 95% confidence interval (CI) as following [23]:

$$ \mathrm{Chao}1 = Aobs 1\kern0.5em  + \left({a}^2/ 2b\right) $$

Where:

*Aobs1* is the total number of observed gp60 allele types; *a* is the number of allele types observed once; and *b* is the number of allele types observed twice. The 95% confidence interval was calculated by bootstrap using Past3 software^e^.

For the calculation, spatial clustering of gp60 alleles was removed by including only one isolate for each allele type present in one region only, and one isolate per each allele type-farm combination [24].

The BLGs of historical human *C. parvum* clinical isolates submitted for genotyping to MU between 2003 and 2010 by diagnostic laboratories were used for comparison with the bovine BLGs. Except for the region of origin, demographic data on human *C. parvum* isolates were not available so it was not possible to account or adjust for the correlation attributable to, for example, lack of independence of outbreak isolates. At the time of this analysis, the BLGs of 298 human *C. parvum* isolates were available.

## Results

### Sample demographics and *C. parvum* prevalence

In total, 1283 calves on 97 farms could eventually be sampled. On each farm the number of collected specimens ranged between 10 and 15. There were 429 and 797 specimens from 1 to 5 and 9 to 21 days-old calves, respectively. Fifty seven specimens arrived without age specification. One hundred and seventy six (13.7%) specimens from 63/97 (65%) farms were IFA-positive. Eighty-four out of 100 IFA-positive genotyped specimens from 55 farms were successfully identified to species-level by 18S rRNA sequencing. *C. parvum* (100% matching to GenBank: AF108864.1) was identified in 77/84 (91.7%) specimens from 49/97 farms (farm-prevalence: 50.5%), and *C. bovis* (100% matching GenBank: KJ531689.10) was found in 7/84 (8.3%) specimens from 6/97 (6.2%) farms. In all the IFA-positive farms multiple parasites belonged to the same species, and on 8/97 (8.2%) farms the species could not be identified as the PCR failed at all three loci.

### Subtyping results

Table 1 shows the distribution of bovine and human BLGs according to their region of origin. All the bovine isolates identified as *C. parvum* by 18 s rRNA sequencing were successfully subtyped at the gp60 and Hsp70 genes. Conversely, parasites identified as *C. bovis* did not show positive amplification at the gp60 and Hsp70, suggesting primer site divergence between the species. Six gp60 alleles were identified in bovine *C. parvum*: the IaA18G3R1, IIaA19G4R1, IIaA16G3R1, and IIaA15G2R1 [100% matching to GenBank: JQ362494.1, JF727803.1, JQ362492.1, JF727755.1, respectively], and a IIaA19G3R1 and IIdA22G1 (deposited by the authors in GenBank: KR052031-KR052032). The Chao1 total richness estimator for bovine gp60 allele types was 6 (95% CI, 4–7), suggesting that the sampling from bovine *C. parvum* was fairly exhaustive and increasing the sampling effort would not have resulted in a significant increase in the number of gp60 allele types observed. Interestingly, 3/15 farms that provided multiple specimens for genotyping displayed more than one gp60 allele. Only three Hsp70 alleles were observed in bovine *C. parvum,* and farms providing multiple specimens displayed a single Hsp70 allele. One Hsp70 allele had 12 GGMP-coding units, and two had 11 units and differed in the synonymous repeat motif (all the Hsp70 alleles found in this study have been deposited in GenBank: KR052026- KR052030). The combinations of gp60 and Hsp70 alleles generated 12 BLGs among 77 bovine *C. parvum*. Conversely, the 298 human *C. parvum* clinical isolates defined 13 gp60 and 5 Hsp70 alleles, which combined defined 20 BLGs. Three out of six bovine *C. parvum* gp60 alleles were represented in the human *C. parvum* sample, as opposed to 10 alleles present in human *C. parvum* and not in cattle. Four gp60 alleles found in humans only were observed in 2010 (Table 1). The BLGs composed of IIaA18G3R1 and IIaA19G4R1 gp60 alleles were the most abundant, accounting for 303/374 (81%) of all the isolates in the dataset, and were ubiquitous and present in both host populations. On the other hand, some BLGs and gp60 alleles appeared host-restricted. For instance, parasites with BLGs containing the IIaA19G3R1 allele were observed only in humans in both islands, and IIdA22G1 alleles were observed in both islands in multiple farms, but only in cattle.

**Table 1:** **The number of** ***Cryptosporidium parvum*** **isolates for each bilocus genotype (BLG) identified in calves (the number of dairy farms is in brackets) and in historic human clinical isolates, sorted by their regions of origin**

**Bilocus genotype designation (gp60/Hsp70 allele)**	**South Island**				**North Island**										**Other regions or unknown location**	**Number of isolates**
	**Canterbury**		**Southland**		**Northland**		**Manawatu–Wanganui**		**Taranaki**		**Waikato**		**Wellington**			
	**Bovine**	**Human**	**Bovine**	**Human**	**Bovine**	**Human**	**Bovine**	**Human**	**Bovine**	**Human**	**Bovine**	**Human**	**Bovine**	**Human**	**Human**	
*IIaA15G2R1/11*	-	-	-	-	-	-	-	-	2 (2)	-	-	-	-	-	-	2
*IIaA16G3R1/11*	-	-	2 (2)	-	-	-	-	-	-	-	1 (1)	-	-	6	4	13
*IIaA18G3R1/11*	1 (1)	5	6 (5)	22	-	-	2 (2)	5	1 (1)	-	22 (14)	3	-	-	30	97
*IIaA18G3R1/12*	1 (1)	-	2 (1)	1	3 (1)	-	2 (1)	2	-	-	1 (1)	36	-	1	60	109
*IIaA18G3R1/12v*	-	-	-	-	-	-	-	-	6 (2)	-	-	-	-	-	-	6
*IIaA18G3R1/12v*_*1*_	-	-	-	-	-	-	-	-	-	-	-	1	-	1	-	2
*IIaA18G3R1/NA*	-	-	-	-	-	-	-	-	2 (1)	-	-	-	-	-	-	2
*IIaA19G4R1/11*	-	-	-	21	-	-	1 (1)	11	4 (3)	4	1 (1)	1	-	2	20	65
*IIaA19G4R1/12*	-	-	-	1	-	-	4(2)	1	-	5	1 (1)	3	-	-	7	22
*IIaA19G3R1/11*	-	-	3 (2)	-	1 (1)	-	-	-	-	-	-	-	-	-	-	4
*IIaA19G3R1/12*	-	-	2 (2)	-	1 (1)	-	-	-	-	-	-	-	-	-	-	3
*IIdA22G1 /11*	-	-	1 (1)	-	-	-	3 (3)	-	-	-	-	-	-	-	-	4
*IIdA22G1 /12*	-	-	-	-	-	-	1 (1)	-	-	-	-	-	-	-	-	1
IIaA14G1R1/11*	-	-	-	-	-	-	-	-	-	-	-	-	-	-	2	2
*IIaA14G1R1/11v*_*2*_	-	-	-	-	-	-	-	1	-	-	-	-	-	-	-	1
*IIaA15G3R1/12*	-	-	-	-	-	-	-	1	-	-	-	-	-	-	1	2
*iiaA17G1/11*	-	-	-	-	-	-	-	-	-	-	-	-	-	1	-	1
*IIaA17G1/11v*_*1*_ *	-	1	-	-	-	-	-	-	-	-	-	-	-	-	-	1
*IIaA18G2R1/11*	-	-	-	2	-	-	-	-	-	-	-	-	-	-	1	3
*IIaA18G4R1/11*	-	-	-	-	-	-	-	-	-	1	-	-	-	-	-	1
*IIaA19G3R1v/11*	-	1	-	2	-	-	-	1	-	-	-	-	-	-	2	6
*IIaA20G42R1/11*	-	-	-	7	-	-	-	-	-	-	-	-	-	-	-	7
*IIaA20G52R1/11*	-	-	-	5	-	-	-	-	-	-	-	-	-	-	-	5
IIaA20G52R1/12*	-	-	-	-	-	-	-	-	-	-	-	-	-	-	5	5
*IIaA21G42R1/11*	-	-	-	-	-	-	-	2	-	-	-	-	-	-	1	3
IIdA23G1R1/11*	-	-	-	1	-	-	-	-	-	-	-	-	-	-	6	7
*IIdA24G1R1/11*															1	1

BLGs are defined using the gp60 nomenclature suggested by Sulaiman et al. [21], followed by the Hsp70 allele designation. Hsp70 alleles are designated by the number of 12-base repeat units (11 or 12) present in the sequence. Subscripts _‘v’, v1, v2_ indicate variant sequences. BLGs containing gp60 alleles which in 2010 were found in humans only are followed by an asterisk.

## Discussion

### Limitations of the study

This work was needed in view of the plethora of phenotypically similar *Cryptosporidium* variants of uncertain pathogenic and zoonotic potential found in recent years in cattle. This study had some limitations. The primers used for the 18 s rRNA gene annealed to *C. hominis*, *C. parvum* and *C. bovis* and we cannot rule out the presence of other species in the PCR-negative farms. These, however, represented only 8% of the farms. Another limitation was the need to combine human sequence data from multiple years due to the unavailability of a large number of isolates from the last trimester of 2011. Finally, although our sampling frame included the majority of the national cattle inventory, the results may not reflect the situation in non-sampled regions.

### Implications of *C. parvum* prevalence results

The laboratory diagnosis of calf cryptosporidiosis is generally undertaken using methods unable to differentiate between *C. parvum* and other species, such as IFA, light microscopy or antigen detection methods. We found a 65% farm-level prevalence of IFA-positive farms, but the estimated *C. parvum* prevalence dropped to 50.5% when assessed by genotyping. Whereas *C. parvum* was, indeed, the most common species found in calves, *C. bovis* and perhaps other non-parvum species were found in ~20% of the IFA-positive farms. This finding is of value to clinicians, as it indicates that phenotypic tests for *C. parvum* offered by veterinary laboratories for the aetiological diagnosis of neonatal calf diarrhoea may have moderate to high positive predictive values. A positive test result will indicate the presence of this species in most cases, but complementary genotyping might be required for specific investigations of severe calf diarrhoea or suspected zoonotic outbreaks.

Studies from other countries have shown variable proportions of *Cryptosporidium* infected farms, but studies applying random sampling criteria and genotyping to which this study can be meaningfully compared were not found in the literature. A farm prevalence of 40% was found in a small scale study in New Zealand carried out without genotyping [25], and a prevalence >76% was reported in the Canadian states of Ontario in a study using a convenience sample of 51 farms without genotyping [26]. Most New Zealand dairy farms manage short and concentrated calving seasons, and we sampled calves during the second half of the season in order to allow the building up of infective oocysts during the first weeks of calving. It is possible that the long interval of time between calving seasons has a sanitising effect, preventing the accumulation of oocysts in the farm environment and establishment of endemic infections in all the farms in successive years. Whether calves on *C. parvum*-negative farms become infected later in life, or cross-immunity to *C. parvum* develops as a consequence of a previous *C. bovis* infection, remains unknown.

### Patterns revealed from *C. parvum* subtyping

The majority of the bovine *C. parvum* BLGs carried gp60 alleles belonging to the IIa allelic family. In particular, BLGs carrying the IIaA18G3R1 and IIaA19G4R1 alleles accounted for the vast majority of the isolates and were the most common variants in both host species and sampled regions (Table 1). In general, parasites carrying IIa alleles have been considered potentially zoonotic, transmissible from livestock [27]. Studies in other countries have reported the IIaA18G3R1 and IIaA19G4R1 in cattle and humans [12,28], and the IIaA18G3R1 was the dominant allele in *C. parvum* from humans, calves, and horses in New Zealand in previous years [21]. There are a number of possible explanations for why parasites carrying the IIaA18G3R1 persist as the most dominant in New Zealand, including a greater infectivity compared with other circulating parasites. On the other hand, some BLGs were host restricted. For instance, BLGs carrying IIdA22G1 alleles were found in cattle in multiple farms in both islands. Whether this restriction reflected host-specificity or limited epidemiological opportunities for the transmission to humans, is not known.

The Chao1 taxonomic richness estimator suggested that the bovine gp60 allelic types have been exhaustively sampled. Thus, the fact that 10 low abundance gp60 alleles were found only in humans (in particular the gp60 alleles found only in humans in 2010 and the IIaA19G3R1, observed in humans in multiple regions; Table 1) suggests that a significant proportion of human infections did not originate from local cattle reservoirs. A similar pattern has been hypothesised in Scotland [24]. It is possible however, that some of these alleles may have been more prevalent in cattle in previous years, within the timeframe of the human sampling.

Finally, the genetic structure of *C. parvum* may be more complex than previously thought, with the ‘isolates’ possibly representing genetic microcosms rather than homogeneous populations of sporozoites [29]. It has been demonstrated that this genetic structure may remain hidden from our observation due to the inability of end point PCR-sequencing to resolve complex DNA mixtures [30]. We observed more than one MLG in 3 out of 15 (20%) farms providing multiple parasites for genotyping. On each farm the calves were sampled on the same day. Thus, it is possible that they carried a range of gp60 alleles, which remained undetected.

## Conclusions

*C. parvum* is the predominant species cycling in newborn dairy calves in New Zealand and is present in ~50% of the farms. The finding of *C. bovis* and perhaps other non-parvum species in a non-negligible proportion of IFA-positive farms indicate that phenotypic tests for *C. parvum* offered by veterinary diagnostic laboratories may have a moderate to high positive predictive values. The genetic similarities between human and bovine *C. parvum* support a model considering the newborn calf population as a significant amplifier of potentially zoonotic parasites. However, data suggest that transmission routes not linked to dairy cattle should also be taken into account in future source-attribution studies of cryptosporidiosis.

## Endnotes

^a^New Zealand Dairy statistics 2010–11. DairyNZ. See: http://www.lic.co.nz/pdf/DAIRY%20STATISTICS%2010-11-WEB.pdf accessed 10 October 2013.

^b^http://www.nzpho.org.nz/NotifiableDisease.aspx; accessed 12 January 2015.

^c^http://freedom.ausvet.com.au/pmwiki/pmwiki.php?n=Freedom.ExactMethod; accessed January 2012.

^d^http://blast.ncbi.nlm.nih.gov/Blast.cgi, accessed in November 2012.

^e^http://folk.uio.no/ohammer/past/; accessed March 2015.
